# Direct comparison of two extended half-life PEGylated recombinant FVIII products: a randomized, crossover pharmacokinetic study in patients with severe hemophilia A

**DOI:** 10.1007/s00277-020-04280-3

**Published:** 2020-09-24

**Authors:** Alexander Solms, Anita Shah, Erik Berntorp, Andreas Tiede, Alfonso Iorio, Camila Linardi, Maurice Ahsman, Maria Elisa Mancuso, Tihomir Zhivkov, Toshko Lissitchkov

**Affiliations:** 1 Clinical Pharmacometrics, Pharmaceuticals Research & Development, Bayer AG, 13353 Berlin, Germany; 2 Bayer, Whippany, NJ USA; 3 Centre for Thrombosis and Haemostasis, Lund University, Skåne University Hospital, Malmö, Sweden; 4 Department of Hematology, Hemostasis, Oncology and Stem Cell Transplantation, Hannover Medical School, Hanover, Germany; 5 McMaster-Bayer Endowed Research Chair in Clinical Epidemiology of Congenital Bleeding Disorders, Department of Medicine, McMaster University, Hamilton, Canada; 6 Department of Health Research Methods, Evidence and Impact, McMaster University, Hamilton, Canada; 7 LAP&P Consultants BV, Leiden, the Netherlands; 8 Center for Thrombosis and Hemorrhagic Diseases, Humanitas Clinical and Research Center – IRCCS, Rozzano, Milan, Italy; 9 Specialized Hospital for Active Treatment, Sofia, Bulgaria

**Keywords:** Factor VIII, Extended half-life, Hemophilia A, PEGylated, Head-to-head study, Population pharmacokinetics

## Abstract

An open-label, crossover randomized study was performed to compare the pharmacokinetics (PK) of damoctocog alfa pegol and rurioctocog alfa pegol, two recombinant factor VIII (FVIII) products indicated in patients with hemophilia A, both conjugated to polyethylene glycol to reduce clearance and extend time in circulation. Adult patients (*N* = 18) with severe hemophilia A (FVIII < 1 IU/dL), previously treated with any FVIII product for ≥ 150 exposure days, were randomized to receive a single 50 IU/kg infusion of damoctocog alfa pegol followed by rurioctocog alfa pegol, or vice versa, with ≥ 7-day washout between doses. FVIII activity was measured using the one-stage clotting assay. PK parameters, including area under the curve from time 0 to the last data point (AUC_0–tlast_, primary parameter), dose-normalized AUC (AUC_norm_), and time to threshold, were calculated based on 11 time points between 0.25 and 120 h post-dose and evaluated using a noncompartmental model. Due to differences in batch-specific vial content used for the study, actual administered median doses were 54.3 IU/kg for damoctocog alfa pegol and 61.4 IU/kg for rurioctocog alfa pegol. Based on actual dosing, a significantly higher geometric mean (coefficient of variation [%CV]) AUC_norm_ was observed for damoctocog alfa pegol (43.8 h kg/dL [44.0]) versus rurioctocog alfa pegol (36.0 h kg/dL [40.1, *P* < 0.001]). Based on population PK modeling, median time to reach 1 IU/dL was 16 h longer for damoctocog alfa pegol compared with rurioctocog alfa pegol. No adverse events or any immunogenicity signals were observed. Overall, damoctocog alfa pegol had a superior PK profile versus rurioctocog alfa pegol. Trial registration number: NCT04015492 (ClinicalTrials.gov identifier). Date of registration: July 9, 2019

**Electronic supplementary material:**

The online version of this article (10.1007/s00277-020-04280-3) contains supplementary material, which is available to authorized users.

## Introduction

In patients with severe hemophilia A (factor VIII [FVIII] levels < 1 IU/dL), prophylaxis with FVIII replacement therapy remains the standard of care [1] and is associated with a reduction in bleeding, including joint bleeds, thereby protecting patients from further complications associated with recurrent bleeds such as joint disease and disability [1, 2]. However, standard replacement recombinant FVIII (rFVIII) products have a short half-life (8–12 h) and, consequently, require frequent infusions to maintain FVIII plasma levels and provide optimal bleeding control [1, 3]. Such frequent dosing is associated with a significant treatment burden and may cause venous access issues or lead to inefficient prophylaxis due to poor patient adherence [4–7].

Extended half-life (EHL) rFVIII products with improved pharmacokinetic (PK) profiles compared with standard half-life (SHL) rFVIII products have the potential to offer longer treatment intervals or to achieve higher trough levels [8, 9]. Covalent attachment of polyethylene glycol (PEG; PEGylation) and fusion with the fragmented crystallizable (Fc) portion of the immunoglobulin (Ig) G1 molecule have been utilized to modify rFVIII to extend its half-life [9, 10].

Damoctocog alfa pegol (BAY 94-9027, Jivi®, Bayer, Germany) and rurioctocog alfa pegol (BAX 855; Adynovate®/Adynovi®; Takeda, Japan) are two EHL rFVIII products that both utilize the attachment of PEG as modification to extend their half-life although the size of the PEG moiety used, site of PEGylation, and FVIII truncation differ between them [11, 12]. Damoctocog alfa pegol is a B-domain-deleted (BDD) rFVIII product that has been site-specifically PEGylated at a single amino acid site with a branched 60 kDa PEG molecule to extend its half-life [11]. Rurioctocog alfa pegol is a full-length rFVIII product that has been PEGylated at its B-domain, with a branched 20-kDa PEG molecule (Advate®; Baxter, USA) [12, 13]. Rurioctocog alfa pegol was first licensed in 2015, and damoctocog alfa pegol in 2018, for the treatment of hemophilia A, based on efficacy and safety data from their respective pivotal phase 2/3 clinical trials [14–19].

Both damoctocog alfa pegol and rurioctocog alfa pegol have demonstrated improvements in their respective PK profiles compared with SHL rFVIII products [17, 20, 21]. In previously treated patients with severe hemophilia A, damoctocog alfa pegol demonstrated an ~ 1.4-fold increase in half-life (*t*_½_) and dose-normalized area under the curve compared with a SHL rFVIII product (sucrose-formulated rFVIII [Kogenate® FS, Bayer, USA]) [20, 21]. For rurioctocog alfa pegol, an ~ 1.4-fold increase in *t*_½_ and an ~ 1.9-fold increase in area under the curve from time 0 to infinity (AUC_*0*–∞_) compared with a conventional rFVIII (Advate®) were confirmed in the phase 2/3 PROLONG-ATE study [17]. Indirect comparisons of damoctocog alfa pegol and rurioctocog alfa pegol using PK data from such studies may be inaccurate and inconclusive, for example, because of interpatient heterogeneity in PK profiles, differences between the studies in the doses and assays used, and in analysis methodologies. Indeed, direct comparisons of PK between different concentrates should ideally be evaluated through a crossover study design to minimize the potential for confounding [22–24].

To date, only one head-to-head crossover PK study of EHL rFVIII products has been reported in hemophilia A. In this study by Shah et al., improvements in several PK parameters following a single infusion of damoctocog alfa pegol were demonstrated in patients with severe hemophilia A, compared with rFVIII Fc fusion protein (rFVIIIFc; Elocta®®; Swedish Orphan Biovitrum Ltd, UK) [25]. In a noncrossover, real-world study conducted in Canada, the PK profiles of rurioctocog alfa pegol and rFVIIIFc were shown to be almost identical in 25 adolescents aged 12–18 years switching from rFVIIIFc to rurioctocog alfa pegol prophylaxis [26]. However, to date, no studies have been performed to compare the PK of two PEGylated EHL rFVIII products. The objective of this study was to directly compare the PK profiles of the two PEGylated EHL rFVIII products, damoctocog alfa pegol and rurioctocog alfa pegol in patients with severe hemophilia A, using a head-to-head, randomized crossover study design. In addition, population PK modeling was performed to explore any differences between damoctocog alfa pegol and rurioctocog alfa pegol in the simulated time to FVIII threshold levels.

## Methods

### Study design

**Fig. 1:**
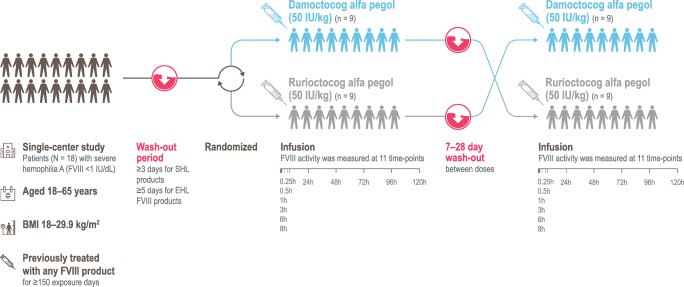
Study design. BMI, body mass index; EHL, extended half-life; FVIII, factor VIII; SHL, standard half-life

This was a single-center, randomized, open-label, single-dose, crossover study (ClinicalTrials.gov identifier: NCT04015492) (Fig. 1). The study was conducted at the National Specialised Hospital for Active Treatment of Haematologic Diseases, in Sofia, Bulgaria. Enrolment began in August 2019 and the last patient to be enrolled completed the study in January 2020. After a washout period of ≥ 3 days or ≥ 5 days for SHL or EHL FVIII products, respectively, patients were randomized 1:1 to receive a single infusion of 50 IU/kg damoctocog alfa pegol or 50 IU/kg rurioctocog alfa pegol, followed by a crossover to a single infusion of the other product, with 7–28-day washout period between doses to account for washout between treatments and patient schedules. Both products were administered as intravenous infusions of up to 10 min. One batch was used for each study drug. Study drug doses were based on the nominal value on the label of the vial. The study was approved by the institutional review board at the single site and was carried out in compliance with the protocol, the principles of the 1975 Declaration of Helsinki and subsequent amendments, and good clinical practice guidelines. All patients gave written informed consent before initiation of any study-related procedures. A patient lay summary can be found in the Supplementary materials (Online resource 1).

### Patients

Eligible patients were males aged 18–65 years with severe hemophilia A (FVIII < 1 IU/dL) previously treated with any FVIII product for ≥ 150 exposure days (EDs). Patients were required to have a body mass index of 18–29.9 kg/m^2^ and were able to stop their current FVIII treatment to complete the wash-out period before study entry and between PK doses. Key exclusion criteria included the presence or history of a FVIII inhibitor (≥ 0.6 Bethesda units/mL), diagnosis of any bleeding disorder other than hemophilia A, platelet count < 75,000/mm^3^, HIV positive with a CD4 count of < 200/mm^3^, serum creatinine over twice the upper limit of normal (ULN), alanine aminotransferase or aspartate aminotransferase over 5 times ULN, or severe liver disease.

### Pharmacokinetic assessments

Plasma samples were collected pre-dose and 0.25, 0.5, 1, 3, 6, 8, 24, 48, 72, 96, and 120 h after infusion of each drug. FVIII levels were measured using the same one-stage clotting assay that was validated for use for both damoctocog alfa pegol and rurioctocog alfa pegol (Online resource 2). Plasma concentrations of damoctocog alfa pegol and rurioctocog alfa pegol were determined by a turbidimetric assay with the SynthASil reagent and activated partial thromboplastin time (APTT) measured on the ACL Advance System against a calibration curve of standard human plasma. The following PK parameters were assessed: AUC from time 0 to the last data point (AUC_0–tlast_; primary PK parameter), AUC; AUC normalized for actual dose per body weight (AUC_norm_), maximum concentration (*C*_max_), normalized *C*_max_ (*C*_max, norm_); *t*_½_; clearance (CL); mean residence time (MRT); volume of distribution at steady state (*V*_ss_); and incremental recovery. The dose-normalized parameters were based on dosing adjusted for actual potency, as per the certificate of analysis for each product provided by the manufacturers. All parameters were calculated using noncompartmental analysis (NCA).

### Population PK model

A single integrated population PK (popPK) model for damoctocog alfa pegol and rurioctocog alfa pegol was developed to simulate “time to reach” FVIII threshold levels of 1, 3, 5, and 10 IU/dL for this study population. The analysis was based on data from all 18 participants.

A nonlinear mixed-effect modeling approach was utilized, as implemented in NONMEM® (version 7.4.1; ICON, Hanover, MD, USA). First, a structural model for each product was selected based on standard diagnostic tools, such as raw-data inspection, goodness of fit, and precision of parameter estimates. As suggested by previous analyses, potential candidates were one- or two-compartment models parameterized in terms of CL and central volume (Vc) and, for the two-compartment model, peripheral volume (Vp) and intercompartmental clearance (*Q*). Residual (unexplained) variability was described using a combined (proportional and additive) error model. Data below the lower limit of quantification (LLOQ) were accounted for using the M3 method [27]. Next, an integrated model was developed by combining the two structural models and subsequently refining the model by testing whether damoctocog alfa pegol and rurioctocog alfa pegol have statistically significant differences in PK parameters (e.g., CL) using the likelihood ratio test (LRT) and a *P* value of 0.01. Because of the small study size, no additional covariate search was conducted. The popPK model was qualified using standard model diagnostic tools, such as uncertainty in parameter estimates, plausibility of estimates (comparison with published information), goodness-of-fit plots, and visual predictive checks.

A sensitivity analysis was performed comparing the results of the approach described above with an alternative approach where previously reported popPK models for damoctocog alfa pegol [28] and rurioctocog alfa pegol [29] were employed (Online resource 3, Online resource Table 1).

### Safety

Safety was assessed by means of clinical and laboratory evaluation at study visits and the recording of adverse events (AEs) and serious (S)AEs. All safety analyses were performed on the safety analysis set defined as those patients who received at least one dose of either damoctocog alfa pegol or rurioctocog alfa pegol. The study investigators reviewed all relevant AEs and SAEs and also assessed the intensity for each of these events. Laboratory analyses to test FVIII inhibitor and anti-PEG antibody development were also performed.

### Statistical analysis

For statistical analysis of the PK parameters obtained by NCA, a log-normal distribution of the parameters was assumed. Log-transformed parameters were analyzed using analysis of variance (ANOVA), including sequence, patient, period, and treatment effects. Based on these analyses, point estimates (least square means), including confidence intervals (CIs, 90% and 95%) for the damoctocog alfa pegol:rurioctocog alfa pegol ratio, were calculated The lower limit of the 90% CI for the ratio exceeding 0.8 would indicate that damoctocog alfa pegol is noninferior to rurioctocog alfa pegol in terms of PK; the lower limit of the 95% CI for the ratio exceeding 1.0 would indicate that damoctocog alfa pegol is superior to rurioctocog alfa pegol. For PK parameters where a low value reflects an improved outcome, such as CL, the lower limit of the 95% CI for the ratio less than 1.0 would indicate that damoctocog alfa pegol is superior to rurioctocog alfa pegol. Safety analyses were descriptive.

## Results

### Patients

A total of 18 patients were randomized and received single doses of damoctocog alfa pegol and rurioctocog alfa pegol; the demographics and baseline characteristics of the patients are provided in Table 1. The median age of patients was 33.5 years.

**Table 1:** Patient demographics and baseline characteristics

Characteristics	Total *N* = 18
Age, years	
Median (range)	33.3 (23, 56)
Mean (SD)	34.2 (9.5)
Race, *n* (%)	
White	18 (100)
BMI, kg/m^2^	
Median (range)	24.45 (18.1, 29.8)
Mean (SD)	23.76 (4.25)
Previous FVIII replacement therapy, *n* (%)	16 (88.9)
On demand	8 (44.4)
Prophylaxis	8 (44.4)
Hemophilic arthropathy	10 (55.6)
Hepatitis viral infections, *n* (%)	13 (72.2)
Chronic hepatitis C	13 (72.2)

*BMI*, body mass index; *FVIII*, factor VIII; *SD*, standard deviation

### Dose adjustment and PK analyses

The 50-IU/kg doses administered in this study were calculated based on the nominal potencies (1000 IU) as provided on the label of the vials which differed from that of the actual potencies, being 1030 IU/vial for damoctocog alfa pegol and 1141 IU/vial for rurioctocog alfa pegol. This resulted in actual administered doses of approximately 3% and 14.1% higher than the planned 50-IU/kg doses for damoctocog alfa pegol and rurioctocog alfa pegol, respectively. In order to accurately compare the PK of the two products, the dose based on actual potency was considered in the analysis of the PK parameters. Based on the actual potencies, the administered median (range) dose was 54.3 IU/kg (51.5, 56.5) for damoctocog alfa pegol and 61.4 IU/kg (57.1, 65.3) for rurioctocog alfa pegol. For AUC_0–tlast_, the geometric mean value was 2311 h IU/dL (percentage of coefficient of variation [%CV], 44.0%; 95% CI, 1880–2850) following a dose of 54.3 IU/kg of damoctocog alfa pegol and 2150 h IU/dL (%CV, 39.6%; 95% CI, 1780–2600) following a dose of 61.4 IU/kg of rurioctocog alfa pegol. Thus, a numerically higher geometric mean AUC_0–tlast_ was observed for damoctocog alfa pegol compared with rurioctocog alfa pegol for an approximately 10% lower actual dose of damoctocog alfa pegol compared with rurioctocog alfa pegol. For AUC_0–tlast_, the geometric least square mean for the damoctocog alfa pegol:rurioctocog alfa pegol ratio was 1.0747 (90% CI, 0.9958–1.1599; 95% CI, 0.9796–1.1790).

**Fig. 2:**
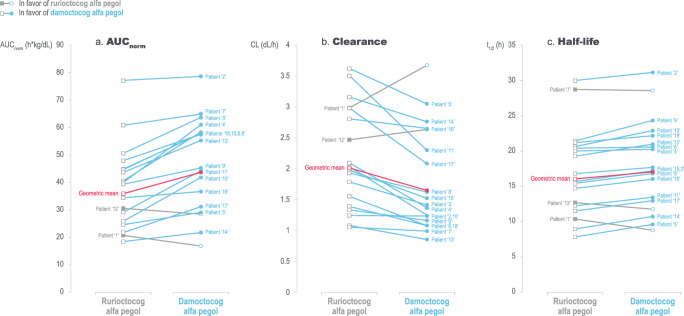
Dose-normalized AUC, clearance, and half-life after a single infusion of damoctocog alfa pegol or rurioctocog alfa pegol. Blue and gray lines indicate those patients who are in favor of damoctocog alfa pegol and rurioctocog alfa pegol, respectively. AUC_norm_, area under the curve normalized for dose per body weight; CL, clearance; *t*_1/2_, half-life

**Fig. 3:**
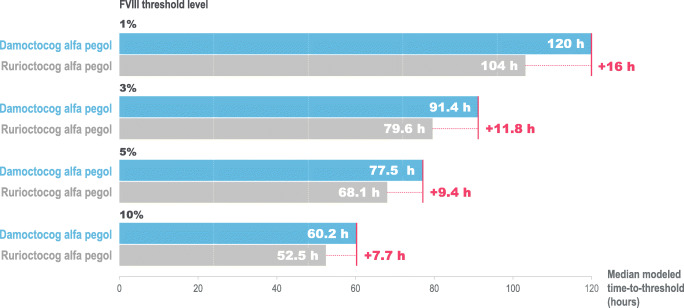
Median modeled time to FVIII threshold level after a single infusion of 50 IU/kg damoctocog alfa pegol or rurioctocog alfa pegol. FVIII, factor VIII; h, hours

Dose-normalized analyses considering the dosing based on actual potency were performed for an accurate and valid comparison of PK parameters between damoctocog alfa pegol and rurioctocog alfa pegol. The geometric mean (%CV) for AUC_norm_ was 43.8 h kg/dL (44.0) for damoctocog alfa pegol and 36.0 h kg/dL (40.1) for rurioctocog alfa pegol (Table 2). Out of 18 patients, 16 (88.9%) showed a higher AUC_norm_ with damoctocog alfa pegol compared with rurioctocog alfa pegol (Fig. 2a). The geometric least square mean for the damoctocog alfa pegol:rurioctocog alfa pegol ratio was 1.22, meeting the prespecified criteria for superiority (95% CI 1.11–1.33, *P =* 0.0004, Table 2). This superiority also corresponded to a significantly slower clearance of damoctocog alfa pegol, resulting in a prolonged *t*_1/2_ versus rurioctocog alfa pegol. CL was significantly reduced for damoctocog alfa pegol compared with rurioctocog alfa pegol (1.65 dL/h, [1.33–2.06] versus 2.01 dL/h, [1.64–2.46]), *P <* 0.001, Table 2), and a reduced clearance with damoctocog alfa pegol compared with rurioctocog alfa pegol was observed in 16 (88.9%) out of 18 patients (Fig. 2b). The geometric mean [%CV, 95% CI] *t*_1/2_ indicated a significantly longer *t*_1/2_ for damoctocog alfa pegol (17.0 h, [37.9%, 14.1–20.4]) versus rurioctocog alfa pegol (16.0 h, [39.0%, 13.2–19.3], *P =* 0.0064, Table 2). The prolonged *t*_1/2_ was observed in 15 (83.3%) out of 18 patients (Fig. 2c). Based on the individual data for all 18 patients, outcomes for AUC_norm_, CL, and *t*_1/2_ were in favor of damoctocog alfa pegol in the majority of patients (Fig. 3). Additional PK parameters are provided in Table 2.

**Table 2:** Dose-normalized PK parameters following single-dose administrations of 54.3 IU/kg of damoctocog alfa pegol and 61.4 IU/kg of rurioctocog alfa pegol

PK parameter	Geometric mean (%CV) (95% CI)		Ratio geometric least square mean ratio^a^ (95% CI)	*P* value	In favor of damoctocog alfa pegol *N* = 18
	Damoctocog alfa pegol	Rurioctocog alfa pegol			
AUC_norm_^b^, kg h/dL	43.8 (44.0) (35.5–54.0)	36.0 (40.1) (29.7–43.7)	1.22 (1.11–1.33)	0.0004	16
*C*_max_^b^, kg/dL	1.99 (24.3) (1.76–2.24)	1.85 (28.6) (1.61–2.12)	1.08 (0.97–1.19)	0.1464	14
CL^b^, dL/h	1.65 (46.4) (1.33–2.06)	2.01 (42.0) (1.64–2.46)	0.82 (0.75–0.90)	0.0004	16
*t*_1/2_, h	17.0 (37.9) (14.1–20.4)	16.0 (39.0) (13.2–19.3)	1.06 (1.02–1.11)	0.0064	15
MRTIV, h	24.59 (37.6) (20.5–29.5)	22.81 (37.1) (19.1–27.3)	1.08 (1.03–1.13)	0.0039	13
*V*_ss_^b^, dL	40.7 (16.2) (37.6–44.1)	45.8 (15.8) (42.4–49.6)	0.89 (0.83–0.95)	0.0024	12
Incremental recovery	1.91 (25.2) (1.69–2.16)	1.71 (33.1) (1.46–2.01)	1.12 (0.98–1.27)	0.0856	14

*AUC*, area under curve from 0 to infinity; *AUC*_*norm*_, AUC normalized for actual dose per body weight; *CL*, clearance; *C*_*max*_, maximum concentration, *C*_*max, norm*_, *C*_max_ normalized for actual dose per body weight; *t*_*1/2*_, half-life; *MRTIV*, mean residence time after injection; *PK*, pharmacokinetic; *V*_*ss*_, volume of distribution at steady state

^a^Ratio of damoctocog alfa pegol:rurioctocog alfa pegol

^b^Based on actual doses (potency-adjusted)

### Population PK modeling and time to threshold simulation

A second distribution compartment could not be identified for damoctocog alfa pegol (CV on *Q* = 103%); the PK of damoctocog alfa pegol was adequately described by a one-compartment model (technically, the PK of damoctocog alfa pegol was described by a two-compartment model fixing *Q* to a very small value [0.001]), while a two-compartment model was used for rurioctocog alfa pegol. Compared with damoctocog alfa pegol, the CL of rurioctocog alfa pegol was significantly increased by 21% (95% CI, 13–29). This effect was consistent across patients with an estimated between-patient variability of 11.5% CV and 17 out of 18 patients having a CL value in favor of damoctocog alfa pegol compared with rurioctocog alfa pegol, respectively. The parameter estimates of the popPK model are shown in Table 3.

**Table 3:** Parameter estimates of the population PK model

PK parameter	Value	SE	CV	95% CI
CL, dL/h	1.63	0.159	9.75	1.32–1.95
V1, dL	39.4	1.26	3.20	36.9–41.9
*Q* rurioctocog alfa pegol, dL/h	3.00	0.726	24.2	1.58–4.43
V2 rurioctocog alfa pegol, dL	5.32	0.817	15.4	3.72–6.92
Relative increase in CL for rurioctocog alfa pegol	1.21	0.0396	3.27	1.13–1.29
Interindividual variability in CL, %CV	41.2	0.0615	36.3	0.0491–0.290
Interindividual variability in V1, %CV	13.0	0.00574	34.1	0.00558–0.0281
Interindividual variability in relative increase in CL for rurioctocog alfa pegol versus damoctocog alfa pegol, %CV	11.5	0.00429	32.4	0.00484–0.0217
Interindividual variability for *Q* rurioctocog alfa pegol, %CV	15.0 (fix)	–	–	–
Interindividual variability for V2 rurioctocog alfa pegol, %CV	15.0 (fix)	–	–	–
Residual error (additive)	0.25 (fix)	–	–	–
Residual error (proportional)	0.117	0.00499	4.26	0.107–0.127

*CI*, confidence interval; *CL*, clearance; *CV*, coefficient of variation; *LBW*, lean body weight; *PK*, pharmacokinetic; *Q*, intercompartmental clearance; *SE*, standard error; *V*, volume

The PK model parameters and results for damoctocog alfa pegol are consistent with previous popPK analyses [25, 29].The median times to reach 1, 3, 5, and 10 IU/dL were 16, 11.8, 9.4, and 7.7 h longer for a dose of 50 IU/kg, respectively, for damoctocog alfa pegol versus rurioctocog alfa pegol (Fig. 3). Sensitivity analyses using published population PK models provided similar results (Online resource 3). This suggests that the data from this study is consistent with PK data described earlier using damoctocog alfa and rurioctocog alfa pegol and that the conclusions from this study are robust with respect to the population PK modeling approach pursued.

### Safety

No adverse events were reported in this study. None of the patients developed antibodies against damoctocog alfa pegol or PEG during the entire study period.

## Discussion

This is the first randomized head-to-head study conducted to directly compare the PK profiles of two PEGylated EHL rFVIII products, damoctocog alfa pegol and rurioctocog alfa pegol, following a single infusion in patients with severe hemophilia A. Owing to the discrepancy in dosing noted in our study (3% and 14% higher than the planned dose of 50 IU/kg for damoctocog alfa pegol and rurioctocog alfa pegol, respectively), analyses based on actual doses were crucial for a valid, direct comparison of the PK profiles of damoctocog alfa pegol and rurioctocog alfa pegol. Taking the actual dosing into consideration, damoctocog alfa pegol demonstrated superiority in PK compared with rurioctocog alfa pegol, with a 22% increase in AUC_norm_ compared with rurioctocog alfa pegol, mirrored by a reduction in the clearance of damoctocog alfa pegol (reduced by 18%), and a prolonged *t*_½_ by 1 h (6% longer) compared with rurioctocog alfa pegol. Importantly, the improvement in PK for damoctocog alfa pegol compared with rurioctocog alfa pegol was observed in most patients in this study.

Improved PK of damoctocog alfa pegol was also observed in a previous head-to-head crossover study, which demonstrated similar levels of improvements in *t*_½_, CL, and AUC for damoctocog alfa pegol compared with rFVIIIFc following a single infusion in patients with severe hemophilia A [25]. The mean AUC_0–tlast_ was 25% higher for damoctocog alfa pegol, with a 20% reduction in CL observed for damoctocog alfa pegol compared with rFVIIIFc. Conducted at the same clinical site and using the same one-stage clotting assay as the current study, similar PK values for damoctocog alfa pegol were also observed with, for example, a *t*_½_ for damoctocog alfa pegol of 16.3 h compared with that reported in the current study (17.0 h). Results from Shah et al. and the current study are indicative of the consistency in improvement of PK parameters for damoctocog alfa pegol, compared with the respective comparator EHL FVIII products rFVIIIFc and rurioctocog alfa pegol. The improvements observed for AUC and CL, translating to prolonged time-to-threshold, also suggest that these PK parameters may be more valuable to consider than the relatively small, albeit significant, differences in half-life reported, when exploring differences in the PK profiles of EHL rFVIII products.

As patients present with varied clinical phenotypes, tailored patient therapy should be guided by careful consideration of the overall improved PK profile of an EHL rFVIII product [24, 30]. Besides half-life, PK parameters such as AUC, CL, and time above FVIII threshold level are important contributors to protection from bleeds and, thus, are of clinical significance [31]. In patients with severe hemophilia A, time spent with very low FVIII levels potentially carries the risk of spontaneous joint bleeds [3]. Additionally, duration of treatment effect has direct implications in dosing frequency and related prophylaxis adherence barriers [32]. In this regard, in the current study, the median time to reach 1 IU/dL was 16 h longer for damoctocog alfa pegol compared with rurioctocog alfa pegol. Taken together, the superior PK profile observed for damoctocog alfa pegol, compared with rurioctocog alfa pegol, in the current study has potential clinical significance in relation to protection from bleeds in patients with hemophilia A. These data suggest patients may benefit from the higher FVIII activity levels and longer time to reach thresholds achieved with damoctocog alfa pegol compared with rurioctocog alfa pegol, using similar doses and dose intervals. Thus, bleed protection can potentially be improved at the same dosing intervals, or existing protection level can be maintained from less frequent infusions with damoctocog alfa pegol.

Properties of the conjugated PEG are known to be driving factors that influence the PK properties of PEGylated proteins [33]. Both the size of the PEG molecule and the type of conjugation are thought to play a role, and it is hypothesized that the differences in these features employed in damoctocog alfa pegol and rurioctocog alfa pegol could contribute to the superior PK profile of damoctocog alfa pegol observed in the current study. Animal models have demonstrated that terminal *t*_1/2_ is affected by PEG size. In hemophilia A mouse and rabbit models, increased conjugated PEG size was associated with an increased terminal *t*_½_: 9.8 h and up to 13.6 h with 30 kDa and 60 kDa PEGylated FVIII, respectively [11]. PEGylation in damoctocog alfa pegol is achieved by a targeted approach of site-specific conjugation at a single site in the BDD rFVIII molecule [11, 34]. Site-specific PEGylation may help to avoid loss of activity or alter protein dynamics due to steric hindrance from extensive PEGylation. The choice of PEGylation site has been shown to influence the activity and molecular dynamics of the conjugated product in other studies [35, 36]. The PEGylation process utilized in rurioctocog alfa pegol, on the other hand, targets the B-domain and thus can introduce PEGylation nonuniformly across the whole length of the B-domain in rFVIII protein [12], including potential unfavorable sites that may affect the molecular dynamics of rurioctocog alfa.

One limitation of the current study was that it was only conducted in adults aged 18–65 years. However, no major differences were observed in the PK profiles between adults and adolescents for damoctocog alfa pegol and rurioctocog alfa pegol [21, 37]. Based on the observation by Shah et al., the improvement in PK observed in this comparison of damoctocog alfa pegol versus rurioctocog alfa pegol should also occur in adolescents [21]. The small size of the patient cohort and the single-center study design could be another potential limitation of this study. However, single-center crossover studies are appropriate for meeting the objectives of this study. A major strength of this study was its crossover design, used to overcome issues around interpatient variability in PK. Intertrial comparison of PK can be inaccurate and inconclusive due to confounding factors such as interpatient variability. Contributing factors for PK variability include interpatient heterogeneity in variables such as von Willebrand factor, age, body size, and blood group [28, 38]. Due to these interpatient variations, a parallel study design or attempts on intertrial comparisons based on previously published data for damoctocog alfa pegol and rurioctocog alfa pegol could result in inaccurate assessment of comparison [22]. The crossover design used in this study thus eliminates the pitfalls associated with confounding factors, as the PK parameters were evaluated using the same assay in the same patient population [22, 23]. Additionally, similarities in the damoctocog alfa pegol PK profile between the current study and the previous head-to-head study by Shah et al. indicate consistency in the modeling process [25]. Another strength of this study was that the data were analyzed using different analysis approaches, including noncompartmental and compartmental methods (popPK). Irrespective of the approach utilized, the findings were the same in that significant differences in PK were detected between damoctocog alfa pegol and rurioctocog alfa pegol, in favor of damoctocog alfa pegol.

In conclusion, damoctocog alfa pegol had a superior PK profile compared with rurioctocog alfa pegol, including a higher AUC_norm_ (based on potency-adjusted analyses), extended half-life, and a longer median time to reach 1% IU/dL FVIII (based on popPK modeling) following a single infusion in patients with severe hemophilia A.

## Electronic supplementary material

ESM 1 (PDF 470 kb)

ESM 2 (DOCX 16 kb)

ESM 3 (DOCX 184 kb)
