# Supplementary material for: Direct comparison of two extended half-life PEGylated recombinant FVIII products: a randomized, crossover pharmacokinetic study in patients with severe hemophilia A
Source: Ann Hematol. 2020 Sep 24;99(11):2689–98. doi: 10.1007/s00277-020-04280-3 (PMC7536163; doi:10.1007/s00277-020-04280-3)
Supplement: Supplementary file 1 — (PDF 470 kb) [file 277_2020_4280_MOESM1_ESM.pdf]

Direct comparison of two extended-half-life PEGylated recombinant FVIII products: a randomized, crossover pharmacokinetic study in patients with severe hemophilia A

Annals of Hematology

Alexander Solms[1] • Anita Shah[2] • Erik Berntorp[3] • Andreas Tiede[4] • Alfonso Iorio[5] • Camila Linardi[2] • Maurice Ahsman[6] • Maria Elisa Mancuso[7] • Tihomir Zhivkov[8] • Toshko Lissitchkov[8]

[1]Bayer, Berlin, Germany; [2]Bayer, Whippany, USA; [3]Centre for Thrombosis and Haemostasis, Lund University, Skåne University Hospital, Malmö, Sweden; [4]Department of Hematology, Hemostasis, Oncology and Stem Cell Transplantation, Hannover Medical School, Hannover, Germany; [5]McMaster-Bayer Endowed Research Chair in Clinical Epidemiology of Congenital Bleeding Disorders, Department of Medicine, and Department of Health Research Methods, Evidence and Impact, McMaster University, Hamilton, Canada; [6]LAP&P Consultants BV, Leiden, the Netherlands; [7]Center for Thrombosis and Hemorrhagic Diseases, Humanitas Clinical and Research Center – IRCCS, Rozzano, Milan, Italy; [8]Specialized Hospital for Active Treatment, Sofia, Bulgaria

Correspondence:

Name:
Alexander Solms

Address:
Bayer AG,
Pharmaceuticals Research & Development,
Clinical Pharmacometrics
13353 Berlin,
Germany

Email:
alexander.solms@bayer.com

A comparison of two similar treatments in people with severe hemophilia A

Official title: **Direct comparison of two extended-half-life PEGylated recombinant FVIII products: a randomized, crossover pharmacokinetic study in patients with severe hemophilia A**

EudraCT trial number: **2018-000507-16**

National clinical trial number: **NCT04015492**

1 What is this study about?

Notes & questions

This is a summary of a clinical study of a medicine called Jivi® (also known as damoctocog alfa pegol) and a medicine called Adynovi® (also known as rurioctocog alfa pegol; Adynovate® in some countries) in people with severe hemophilia A. It is written for the general reader and uses language that is easy to understand. It includes information about how researchers did the study and what the results were.

2 Who sponsored the study and how can I contact them?

The sponsor of this study was Bayer who would like to thank everyone who took part. Their participation helped researchers answer important questions about Jivi® and Adynovi® in people with hemophilia A.

Bayer Pharmaceuticals
Bayer AG
13353 Berlin
Germany

3 When and where did this study take place?

Notes & questions

This study started in August 2019 and finished in January 2020.

It took place in Bulgaria, at the National Specialised Hospital for Active Treatment of Haematologic Diseases.

4 Why was this study done?

Researchers are looking for a better way to treat people with hemophilia A.

Hemophilia A is a form of hemophilia that is due to deficiency of one of the proteins the body produces to clot the blood. This protein is called Factor Eight (FVIII). A deficiency of FVIII can lead to frequent, spontaneous bleeding, often into joints and muscles, in addition to abnormal bleeding following an injury, surgery, or tooth extraction.

Uncontrolled, frequent bleeding could develop into further complications such as joint disease or disability. Therefore, people with hemophilia A are given medicines to prevent bleeding. These medicines replace the FVIII that is missing in these people.

In this study, the main question the researchers wanted to answer was: Is there a difference between the concentration of FVIII in the blood after treatment with Jivi® compared with Adynovi®, over a certain period of time? The answer to this question was important to better understand how Jivi® can improve the health of people with hemophilia A.

5 Who took part in this study?

People who participated in this study had severe hemophilia A and could take part if they were:

- 18–65 years old
- Had a body mass index of 18–29.9 kg/m^2

Overall, 18 people took part in the study and all of them were men. The average age of all the participants was 33.5 years.

All 18 participants completed the study.

6 What treatments did the participants in this study receive?

Notes & questions

The researchers compared two medicines called Jivi® and Adynovi®. Both of these medicines are 'extended-half-life' medicines. This means that they are designed to work for longer than a medicine that isn't designed to have an 'extended-half-life'.

In this study, each participant received both medicines once, one followed by the other.

Adynovi® was chosen as the comparator because it is similar to Jivi® and the two have not been directly compared before.

7 What happened during the study?

- The 18 participants in this study were put into two groups by chance (randomized)
- One group received a single injection of Jivi® first, followed by a single injection of Adynovi®, as described below
- The second group received a single injection of Adynovi® first, followed by a single injection of Jivi®, as described below
- As soon as a participant received their first treatment, samples of blood were taken repeatedly over 5 days to measure the concentration of FVIII in their blood
- After these 5 days, each participant waited a short time before they received their second treatment
- As soon as a participant received their second treatment, samples of blood were taken repeatedly over 5 days to measure the concentration of the FVIII in their blood
- Everyone who took part in the study was also closely monitored for any medical problems
- The participants and the researchers both knew the order in which the medicines were taken
- Except for the difference in the order the medicines were taken, all participants followed the same procedures.

The following diagram shows how the study was done:

Notes & questions

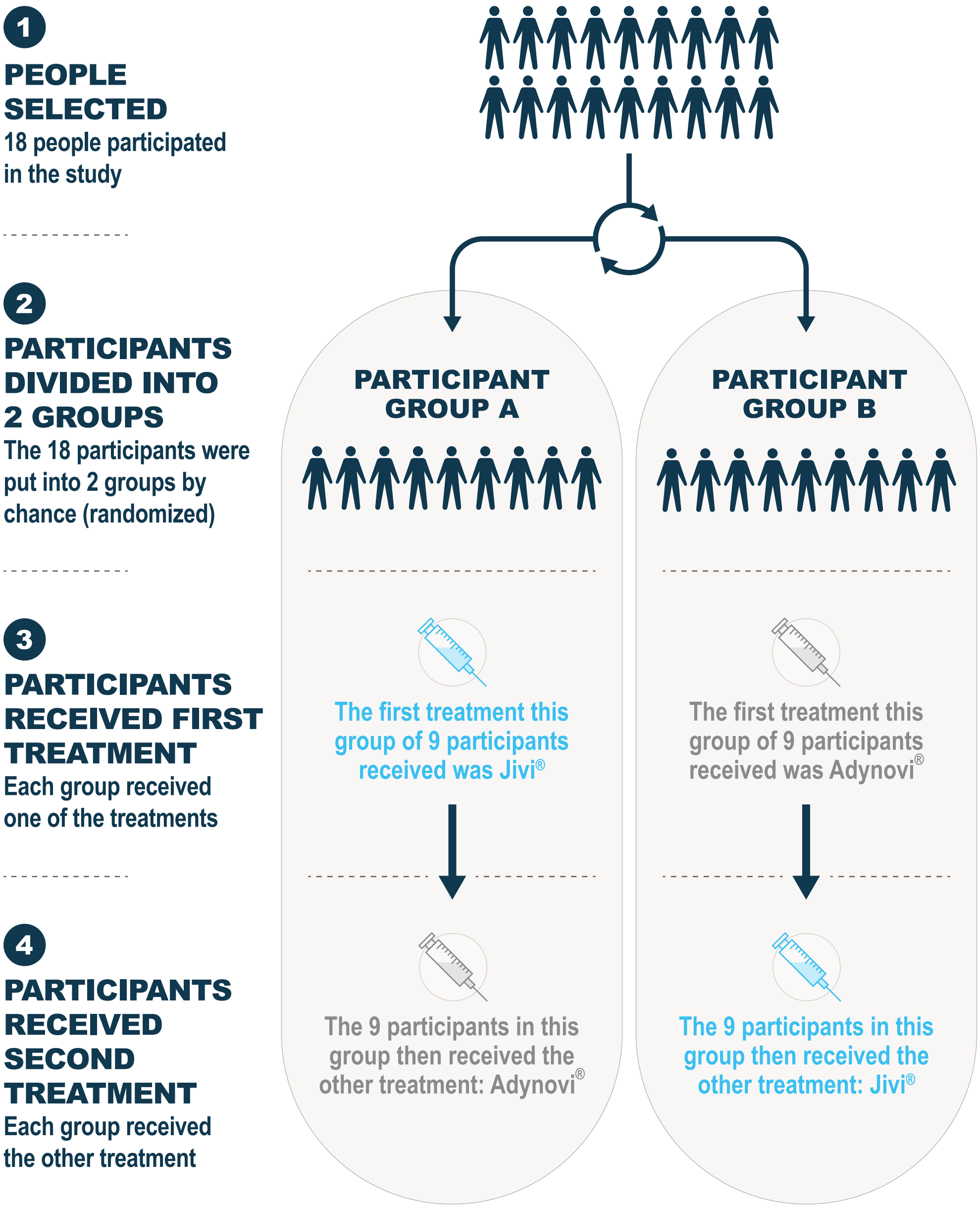

8 What were the results of the study?

Overall, this study showed that there are differences in the concentration of FVIII in the blood after treatment with Jivi® compared with Adynovi®.

In this study, the concentration of FVIII over 5 days after treatment with each medicine was evaluated in different ways.

Notes & questions

1. The researchers measured the amount (exposure) of FVIII protein in the blood over a period of time for each medicine (this is called ‘area under the curve’). Generally, **it is better for the exposure of a medicine to be higher** for it to be available for clotting over time.

In **16** of the 18 participants

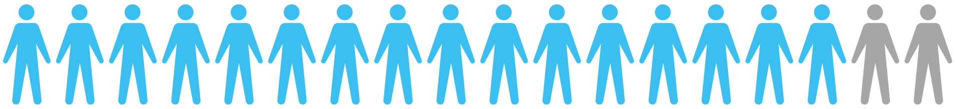

the amount of Jivi® was higher than the amount of Adynovi®

2. The researchers also measured the speed at which each medicine was removed from the body (this is called ‘clearance’). Generally, **it is better for a medicine to be removed slowly** so the medicine can act longer within the body.

In **16** of the 18 participants

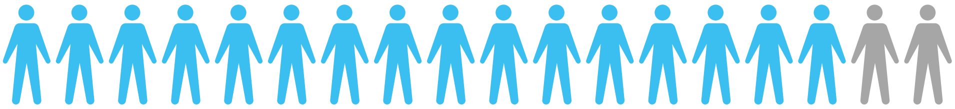

Jivi® was removed from the body more slowly than Adynovi®

3. The researchers also measured the time taken for the concentration of each medicine to be reduced by half in the blood (this is called ‘half-life’). This also helps in assessing how long each medicine remains active in the body. Generally, **it is better for a medicine to remain active for a long period** for it to be available for clotting.

In **15** of the 18 participants

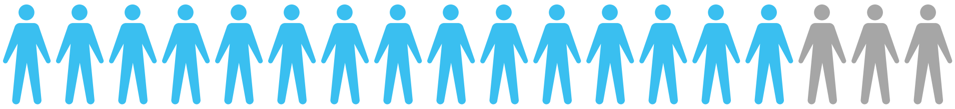

Jivi® remained active in the body for longer than Adynovi®

4. And the researchers calculated the time it took for various concentrations of FVIII for each medicine in the blood to decline to a level at which there is an increased risk of bleeding. This level is different for each person with hemophilia A and can also vary within a person, for instance depending on physical activity. **It is better for the time it takes to reach this level to be longer**, so that people with hemophilia A might need less frequent injections without increasing the risk of bleeding.

Notes & questions

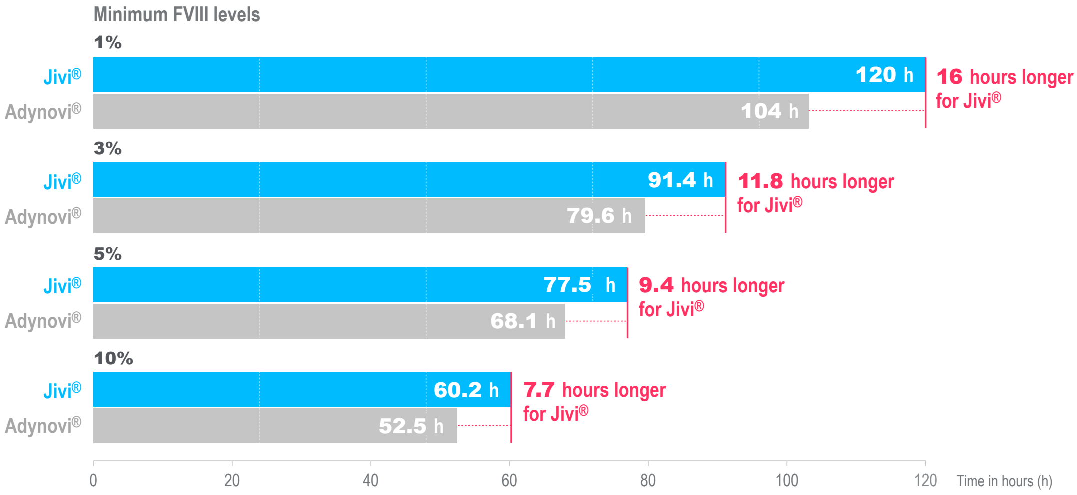

It is important to know the following points about the study results:

- These results are only for this clinical study, which looked at a sample of 18 people with severe hemophilia A
- Not all who participated in the study had the same results
- The results for any individual participant could have been different to the overall results
- These results are not an explanation of what a treatment can and cannot do for an individual
- Generally, a single clinical study cannot give a complete picture of the benefits and risks of a medicine
- Other studies may find different results.

9 What medical problems did the people who took part in the study have?

Notes & questions

Researchers keep track of all health problems that participants have during a study. Some of these health problems might be caused by the study medicines and some by other medicines taken by the participant. These health problems are called side-effects. Other health problems might be caused by the disease, and some could have a yet different cause.

The researchers monitored the safety of Jivi® and Adynovi® in those who took part in this study. To do this, the researchers performed laboratory tests and made notes of any side-effects during the study:

- **None of the participants** had any side-effects from Jivi®
- **None of the participants** had abnormal laboratory test results from Jivi®
- **None of the participants** had any side-effects from Adynovi®
- **None of the participants** had abnormal laboratory test results from Adynovi®.

10 How can this study help people?

This research may help people with hemophilia A and their families by helping doctors understand more about how Jivi® may improve bleeding control which has profound effects on their daily lives.

The study showed that there are differences in the concentration of FVIII in the blood after treatment with Jivi® compared with Adynovi®. These results can help doctors decide if Jivi® is a suitable option for people living with hemophilia A.

11 Are further studies planned?

No further studies on this topic are planned.

12 Where can I learn more about this study?

Notes & questions

Bayer has committed to make research results available to the public. This summary has been provided as part of that commitment and should not be used for any other purpose. It should not be considered to make a claim for any product or to guide treatment decisions.

If you participated in this study and have questions about the study results, the doctor or staff at the study center you attended may be able to answer them.

13 Important notice

This summary shows only the results from one study and does not represent all of the knowledge about the medicines studied. Usually, more than one study is done to find out how well a medicine works and discover the side-effects of the medicine. Other studies may have different results.

You should consult the prescribing information for your country to get more information on the medicines studied or ask your physician about these. You should not change your therapy based on the results of this study without first talking to your physician. Always consult your physician about your specific therapy.
