# Supplementary material for: Direct comparison of two extended half-life PEGylated recombinant FVIII products: a randomized, crossover pharmacokinetic study in patients with severe hemophilia A
Source: Ann Hematol. 2020 Sep 24;99(11):2689–98. doi: 10.1007/s00277-020-04280-3 (PMC7536163; doi:10.1007/s00277-020-04280-3)
Supplement: Supplementary file 2 — (DOCX 16 kb) [file 277_2020_4280_MOESM2_ESM.docx]

**Direct comparison of two extended-half-life PEGylated recombinant FVIII products: a randomized, crossover pharmacokinetic study in patients with severe hemophilia A**

Alexander Solms^1^, Anita Shah^2^, Erik Berntorp^3^, Andreas Tiede^4^, Alfonso Iorio^5^, Camila Linardi^2^, Maurice Ahsman^6^, Maria Elisa Mancuso^7^, Tihomir Zhivkov^8^, Toshko Lissitchkov^8^

^1^Bayer, Berlin, Germany; ^2^Bayer, Whippany, USA; ^3^Centre for Thrombosis and Haemostasis, Lund University, Skåne University Hospital, Malmö, Sweden; ^4^Department of Hematology, Hemostasis, Oncology and Stem Cell Transplantation, Hannover Medical School, Hannover, Germany; ^5^McMaster-Bayer Endowed Research Chair in Clinical Epidemiology of Congenital Bleeding Disorders, Department of Medicine, and Department of Health Research Methods, Evidence and Impact, McMaster University, Hamilton, Canada; ^6^LAP&P Consultants BV, Leiden, the Netherlands; ^7^Center for Thrombosis and Hemorrhagic Diseases, Humanitas Clinical and Research Center – IRCCS, Rozzano, Milan, Italy; ^8^Specialized Hospital for Active Treatment, Sofia, Bulgaria

Correspondence:

Name: Alexander Solms

Address: Bayer AG,

Pharmaceuticals Research & Development,

Clinical Pharmacometrics

13353 Berlin,

Germany

Email: [alexander.solms@bayer.com](mailto:alexander.solms@bayer.com)

ORCID: 0000-0002-0945-2543

Journal: *Annals of Hematology*

## Online resource 2: Validation of one-stage clotting assay used to determine FVIII levels for both damoctocog alfa pegol and rurioctocog alfa pegol

The method validation and analysis of the study samples were performed in compliance with the pertinent guidelines on Bioanalytical Method Validation (FDA 2018; EMA 2011; EMA 2012).

For damoctocog alfa pegol, the calibration range of the procedure was from 1.00 (LLOQ) to 80.0 IU/dL (ULOQ). Mean inter-assay accuracy of back-calculated concentrations (except LLOQ) in calibrators ranged between 98.0% and 102% and precision was ≤ 2.39%. Accuracy and precision at the lowest calibrator (LLOQ) was equal to 99.6% and 0.74%, respectively. Quality control (QC) samples in the concentration range from 2.50 to 60.0 IU/dL were determined with an accuracy of 90.0% to 98.6% and a precision of 8.61% to 10.1%.

For rurioctocog alfa pegol, the calibration range of the procedure was from 1.00 (LLOQ) to 80.0 IU/dL (ULOQ). Mean inter-assay accuracy of back-calculated concentrations (except LLOQ) in calibrators ranged between 98.2% and 102% and precision was
≤ 1.86%. Accuracy and precision at the lowest calibrator (LLOQ) was equal to 99.9% and 0.65%, respectively. Quality control (QC) samples in the concentration range from 2.50 to 60.0 IU/dL were determined with an accuracy of 98.3% to 108% and a precision of 4.95% to 7.14%.

## References

U.S. Department of Health and Human Services Food and Drug Administration Center for Drug Evaluation and Research (CDER) Center for Veterinary Medicine (CVM): Bioanalytical Method Validation Guidance for Industry 2018.

European Medicines Agency: Guideline on bioanalytical method validation.2011.

European Medicines Agency: Reflection paper for laboratories that perform the analysis or evaluation of clinical trial samples. 2012.
