# Supplementary material for: Direct comparison of two extended half-life PEGylated recombinant FVIII products: a randomized, crossover pharmacokinetic study in patients with severe hemophilia A
Source: Ann Hematol. 2020 Sep 24;99(11):2689–98. doi: 10.1007/s00277-020-04280-3 (PMC7536163; doi:10.1007/s00277-020-04280-3)
Supplement: Supplementary file 3 — (DOCX 184 kb) [file 277_2020_4280_MOESM3_ESM.docx]

**Direct comparison of two extended-half-life PEGylated recombinant FVIII products: a randomized, crossover pharmacokinetic study in patients with severe hemophilia A**

Alexander Solms^1^, Anita Shah^2^, Erik Berntorp^3^, Andreas Tiede^4^, Alfonso Iorio^5^, Camila Linardi^2^, Maurice Ahsman^6^, Maria Elisa Mancuso^7^, Tihomir Zhivkov^8^, Toshko Lissitchkov^8^

^1^Bayer, Berlin, Germany; ^2^Bayer, Whippany, USA; ^3^Centre for Thrombosis and Haemostasis, Lund University, Skåne University Hospital, Malmö, Sweden; ^4^Department of Hematology, Hemostasis, Oncology and Stem Cell Transplantation, Hannover Medical School, Hannover, Germany; ^5^McMaster-Bayer Endowed Research Chair in Clinical Epidemiology of Congenital Bleeding Disorders, Department of Medicine, and Department of Health Research Methods, Evidence and Impact, McMaster University, Hamilton, Canada; ^6^LAP&P Consultants BV, Leiden, the Netherlands; ^7^Center for Thrombosis and Hemorrhagic Diseases, Humanitas Clinical and Research Center – IRCCS, Rozzano, Milan, Italy; ^8^Specialized Hospital for Active Treatment, Sofia, Bulgaria

Correspondence:

Name: Alexander Solms

Address: Bayer AG,

Pharmaceuticals Research & Development,

Clinical Pharmacometrics

13353 Berlin,

Germany

Email: [alexander.solms@bayer.com](mailto:alexander.solms@bayer.com)

ORCID: 0000-0002-0945-2543

Journal: *Annals of Hematology*

## Online resource 3: Sensitivity analysis for time to threshold data generated using different population PK (popPK) models

A sensitivity analysis was performed to assess the impact of the choice of the population PK model employed to determine the time to threshold. To this aim, referring to the Bayesian forecasting approach by the WAPPS Hemo PK tool (Iorio et al 2016, Hajducek 2020), the population PK models for damoctocog alfa pegol (Solms et al 2019, based on n=198 patients) and rurioctocog alfa pegol (Chelle et al 2020, based on n=154 patients), for one-stage assay data, were used to derive individual posthoc estimates. Single profiles of damoctocog alfa pegol and rurioctocog alfa pegol for each patient from this study were used for this posthoc estimation. The posthoc estimates were used to predict the time-to-threshold for different thresholds concentrations of 1, 3, 5 and 10 IU/dL for a dose of 50 IU/kg.

For both products, using the published models lead to nearly identical individual time to threshold estimates as the newly developed H2H model across patients, as indicated by a close to 1 coefficient of determination (R^2^). As a result, also only minor differences in median time to threshold were observed depending on which model was used.

**Supplementary Table 1: Sensitivity analysis of time to threshold levels using different population PK modeling approaches.**


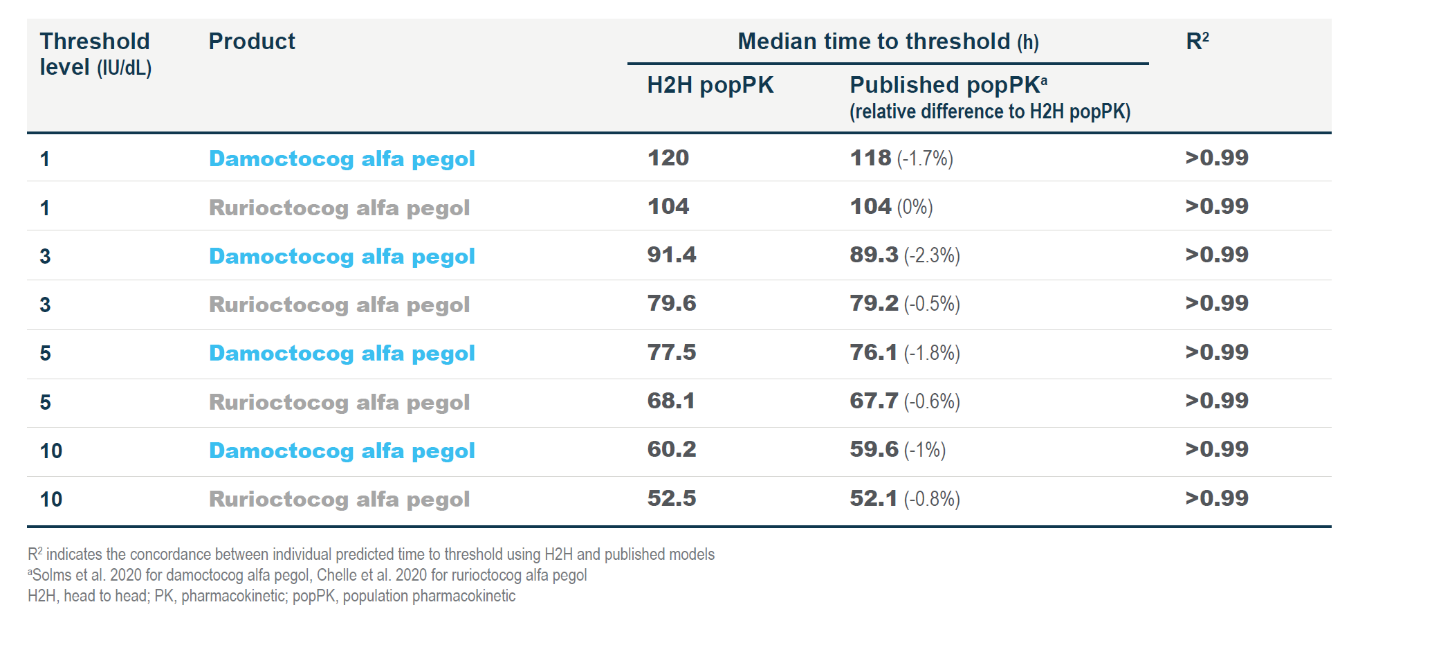


## References

Iorio A, Keepanasseril A, Foster G, et al. Development of a Web-Accessible Population Pharmacokinetic Service-Hemophilia (WAPPS-Hemo): Study Protocol. *JMIR Res Protoc*. 2016;5(4):e239. doi:10.2196/resprot.6558

Hajducek DM, Chelle P, Hermans C, et al. Development and evaluation of the population pharmacokinetic models for FVIII and FIX concentrates of the WAPPS-Hemo project. *Haemophilia*. 2020;26(3):384‐400. doi:10.1111/hae.13977

Solms, A., A. Iorio, M. J. Ahsman, P. Vis, A. Shah, E. Berntorp and D. Garmann (2020). "Favorable Pharmacokinetic Characteristics of Extended-Half-Life Recombinant Factor VIII BAY 94-9027 Enable Robust Individual Profiling Using a Population Pharmacokinetic Approach." Clinical Pharmacokinetics 59(5): 605-616.

Chelle P, Yeung CHT, Croteau SE, et al. Development and Validation of a Population-Pharmacokinetic Model for Rurioctacog Alfa Pegol (Adynovate^®^): A Report on Behalf of the WAPPS-Hemo Investigators Ad Hoc Subgroup. Clin Pharmacokinet. 2020;59(2):245‐256.
